# The Lead Line of the Gums

**Published:** 1876-08

**Authors:** C. Hilton Fagge


### ARTICLE VI.

### *The Lead Line of the Gums.*

Some further facts on this diagnostic mark were communicated last month to the Royal Medical and Chirurgical Society in a paper by Dr. C. Hilton Fagge :

The author had in two cases investigated the microscopical characters of the gingival tissues when affected with the lead-line. He found that the discoloration was not uniform but was distributed in the form of rounded loops, corresponding with the vascular papillæ of the mucous membrane. Under a higher power, the pigment was seen to consist of granules, some of which were deposited in the interior of the smallest blood vessels, others immediately outside them. Mr. Tomes' hypothesis was that the pigment was a sulphuret of lead; and that the sulphuretted hydrogen which formed it was derived from decomposing animal matters which had been retained in the tartar or between the teeth. The author pointed out that the gas must diffuse itself into the texture of the gum and combine with the lead, as it was actually circulating in the blood, or contained in the plasma which was for the nourishment of the textures. The pigment was thus a precipitate from the blood. This view explained the fact that a characteristic line in the gums was sometimes in those who had not shown symptoms of lead poisoning. When occurring under such circumstances, it had been supposed by certain writers to have been caused by the presence of some other metal, such as bismuth. The author thought that it depended on the very minute quantity of lead which had in such cases gained entrance into the body. Another point explicable upon the author's theory of the lead line was that the administration of iodide of potassium sometimes brought it out, when it had before been absent, and after the individual had ceased to be exposed to the poison. It also followed that the line must be wanting in those who kept the teeth very clean. Lastly, it sesmed probable that the sulphide of lead must be deposited in the tntestinal mucous membrane, likewise under the influence of the sulphuretted hydrogen contained in the bowels.—*Med. and Surg. Reporter.*

